# Fermentation of Whole Grain Sorghum (*Sorghum bicolor* (L.) Moench) with Different Dry Matter Concentrations: Effect on the Apparent Total Tract Digestibility of Energy, Crude Nutrients and Minerals in Growing Pigs

**DOI:** 10.3390/ani11051199

**Published:** 2021-04-22

**Authors:** Reinhard Puntigam, Julia Slama, Daniel Brugger, Karin Leitner, Karl Schedle, Gabriela Wetscherek-Seipelt, Wolfgang Wetscherek

**Affiliations:** 1 Faculty of Agricultural and Environmental Sciences, University Rostock, 18059 Rostock, Germany; julia.slama@uni-rostock.de; 2 Institute of Animal Nutrition, Vetsuisse-Faculty, University of Zurich, 8057 Zurich, Switzerland; dbrugger@nutrivet.uzh.ch; 3 Institute of Animal Nutrition, Livestock Products, and Nutrition Physiology, University of Natural Resources and Life Sciences, 1190 Vienna, Austria; karin.leitner@schaumann.at (K.L.); karl.schedle@boku.ac.at (K.S.); gabriela.wetscherek-seipelt@boku.ac.at (G.W.-S.); wolfgang.wetscherek@boku.ac.at (W.W.)

**Keywords:** pig, digestibility, minerals, fermentation, sorghum

## Abstract

**Simple Summary:**

Due to climate change and pests as result of maize monoculture, the need of diversification of crop rotation forces researchers to look for alternative grains for animal nutrition. Furthermore, grain fermentation may increase the nutritional value of feed and, simultaneously, decrease costs of feed conservation because the necessity for grain drying and associated energy costs are reduced. In this context, the cultivation and integration of early harvested and fermented sorghum grain in pig diets might be an interesting strategy for the substitution of maize. Therefore, we tested the nutritional value of three varieties of fermented sorghum grains with gradual differences in total dry matter, in a Latin-Square experiment comprising growing pigs. Results indicated there is a potential for improving the nutrient digestibility of sorghum-based pig diets by using early harvested and fermented whole sorghum grain with lower dry matter concentration. Especially the need for inorganic phosphorus supplementation and, hence, the fecal phosphorus emissions were significantly reduced.

**Abstract:**

This study investigated the effects of sorghum ensiled as whole grains with different dry matter concentrations on the apparent total tract digestibility (ATTD) of energy, crude nutrients and minerals in growing pigs. Whole grain sorghum batches with varying dry matter (DM) concentrations of 701 (S1), 738 (S2) and 809 g kg^−1^ (S3) due to different dates of harvest from the same arable plot, were stored in air-tight kegs (6 L) for 6 months to ensure complete fermentation. Subsequently, 9 crossbred barrows (34.6 ± 1.8 kg; (Duroc x Landrace) × Piétrain)) were used in a 3 × 3 Latin square feeding experiment. Diets were based on the respective sorghum grain silage and were supplemented with additional amino acids, minerals and vitamins to meet or exceed published feeding recommendations for growing pigs. The ATTD of gross energy, dry matter, organic matter, nitrogen-free extracts, and crude ash were higher in S1 compared to S3 treatments (*p* ≤ 0.05), while S2 was intermediate. Pigs fed S1 showed significantly higher ATTD of phosphorus (P) compared to all other groups while ATTD of calcium was unaffected irrespective of the feeding regime. In conclusion, growing pigs used whole grain sorghum fermented with a DM concentration of 701 g kg^−1^ (S1) most efficiently. In particular, the addition of inorganic P could have been reduced by 0.39 g kg^−1^ DM when using this silage compared to the variant with the highest DM value (809 g kg^−1^).

## 1. Introduction

Sorghum represents the fifth most important grain worldwide and could replace significant amounts of maize in poultry and pig diets [1,2,3]. The nutritional composition of sorghum is very similar to maize, while it is, from a botanical point of view, more drought-resistant, has lower demands on soil quality and is not affected by pests like the western corn rootworm (*Diabrotica virgifera virgifera*; [4,5]. Sorghum as a substitution of maize may contribute to the diversification of crop rotation, resulting in reduced maize monocultures and may therefore positively affect the agricultural ecosystem. In addition to phytate, sorghum can also contain high amounts of kafirin and condensed tannins, which may negatively influence its utilization within the gastrointestinal tract [6]. However, European sorghum varieties have been cultivated to be low in tannins over the last 30 years. In fact, it is not possible to register new sorghum varieties unless their tannin concentration is lower than 30 g kg^−1^ [7].

The nutritional value of sorghum grains can be improved through the application of different processing methods, such as grinding [8], hydrothermal processing [9], germination [10] or ensiling [11,12,13]. In some regions in Europe (e.g., Austria, Germany, Slovakia), conserving maize through ensiling is well established because it has a lot of advantages [13,14,15]. Fermentation conservation of high moisture whole grain eliminates the necessity for drying, thereby reducing costs of feed preparation and the risk of degradation of heat labile nutrients [16]. Furthermore, it is well documented that a late harvest date increases the probability of higher mycotoxin concentrations, which may negatively affect feed quality and animal performance. For example, Reid and Sinha [17] and Lauren et al. [18] reported an increase in deoxynivalenol and zearalenone contamination following a delay in the harvest of sorghum cultivars. Since fermentation allows for an earlier harvest of sorghum kernels, it may therefore also exert secondary effects on the hygienic status of monogastric diets.

Grain fermentation has been shown to improve nutrient digestibility in pigs [12,13,19,20,21]. Carlson and Poulsen [22] evaluated the effect of soaked and fermented liquid feed (barley vs. wheat) based on the parameters: diet, time of soaking, heat treatment, phytase activity, pH value, and temperature. They observed significant phytate degradation when grain-based feeds were soaked and fermented before being fed to pigs. In addition, Humer et al. [12] demonstrated that the dry matter (DM) content of grain affects the fermentation intensity and subsequent digestibility of nutrients from ensiled maize kernels in pig diets.

Data on the use of fermented sorghum kernels in pig nutrition are currently scarce, especially with regard to the possible effects of the sorghum DM concentration at the time of harvest. Therefore, the aim of the present study was to investigate the effects of fermented whole grain sorghum with varying DM concentrations on the apparent total tract digestibility (ATTD) of crude nutrients, energy and minerals in growing pigs.

## 2. Materials and Methods

### 2.1. Sorghum Harvest and Fermentation Procedure

The varying DM concentration of sorghum grains (Targga, RAGT, Rodez, France) was achieved by different harvesting dates from the same field (Southeast Styria, Hatzendorf, Austria). Sorghum S1 was harvested on September 24th 2016, while S2 and S3 were harvested 8 and 16 days later, respectively. The varying harvest dates resulted in DM concentrations of 701 (S1), 738 (S2) and 809 g kg^−1^ (S3), respectively. The whole grain sorghum batches were filled in air-tight 6 L wide-neck-kegs (Bär, Salzburg, Austria) and stored protected from light at ambient temperature conditions for 6 months to ensure sufficient fermentation. No silage additives, like microbial inoculants, were used for the production of sorghum grain silage.

### 2.2. Animals and Diets

The protocol of the pig study was approved by the Austrian Ministry for Science and Research and by the University of Natural Resources and Life Sciences, Vienna (BMWF-60016/4-WF/V3b/2015).

The experiment was conducted following a 3 × 3 Latin square design with 9 crossbred barrows, which were progeny of (Duroc × Landrace) × Piétrain. The pigs were randomly allocated to individual metabolic cages (Ehret, Tulln, Austria) according to body weight (BW; 34.6 ± 1.8 kg) and litter (pigs from 3 different litters were used). The stainless-steel cages were equipped with wire mesh screens and drain pans for the separate quantitative collection of feces and urine. The cage size was constantly adjusted to the changing body size of each individual animal during the feeding trial. The barrows were fed equal rations twice per day at 7 AM and 6 PM. Feed intake was restricted to 2.5-fold the maintenance requirement for metabolizable energy (ME) [23], based on the pigs individual life weights at the start of each of three experimental runs. The digestibility experiment started with a 4-day adaptation period to the metabolism cages, during which a commercial diet for growing pigs based on maize and soybean meal was fed ad libitum. Each experimental period consisted of 12 days, with 7 days of diet adaptation and 5 days of sampling period. Thereby, each pig received each type of experimental diet once, respectively, and hence served as its own internal control (*n* = 9 replicates per treatment group). The pigs had free access to tap drinking water throughout the study.

For each new day of experimental feeding, respective sorghum fermentation kegs were freshly opened, and their contents were briefly grounded with a conventional hammer mill (sieve: 4.5 mm, Super 40, Gruber Maschinen GmbH, Gaspoltshofen, Austria) before mixing and feeding the diets, respectively. Table 1 shows the dietary ingredients and chemical composition. The basal diet was formulated according to the recommendations of the GfE [23] except for crude protein and was based on nutrient analysis of the sorghum conserves prior to feed preparation. Thereby, the recommended and comparable crude protein concentrations after inclusion of respective sorghum variants into experimental diets were achieved. Diets consisted of fermented sorghum fortified with crystalline amino acids (AA) and a mineral-vitamin premix. Due to the differences in DM concentrations of sorghum conserves S1, S2 and S3, varying amounts of conserved sorghum were mixed with the premix. To avoid excessive endogenous phosphorus (P) excretion and a bias of P digestibility values arising thereof, no inorganic P was supplemented. As a result, the dietary design met the guidelines of GfE [24] for determination of ATTD of P in pigs.

### 2.3. Sampling

Pooled samples of 3 sorghum conserves were taken at the beginning of each experimental period, vacuum sealed and stored at −20 °C. Individual feces samples were collected quantitatively per day over a period of five consecutive days per experimental run (3 runs with nine animals in total). Fecal batches were weighted on each day of the experimental phase, homogenized and aliquot amounts were stored at −20 °C until further usage. Another aliquot was used for immediate determination of fecal DM according to standard procedures [25].

### 2.4. Analytical Methods

Frozen fecal samples were defrosted, dried under mild conditions (50 °C) and milled (1.0 mm, Retsch ZM200, Haan, Germany) prior to chemical analysis. Milled feeds and feces were subject to analyses of DM, organic matter (OM), crude ash (Ash), crude protein (CP), crude fiber (CF), starch and ether extract after acid hydrolysis (EE_h_) according to standard procedures [25]. Phosphorus was determined photometrically (U5100-Spectrophotometer-Hitatchi, Metrohm, Vienna, Austria) using the vanado-molybdate method to measure color intensity at 436 nm and calcium (Ca) was determined by flame atomic absorption spectrophotometry (AAnalyst 200, Perkin Elmer, Brunn am Gebirge, Austria) following nitric acid wet digestion via microwave (MLS-ETHOS plus Terminal 320, Leutkirch, Germany). Gross energy (GE) contents were determined by bomb calorimetry (IKA-Kalorimeter C400, Bartelt, Graz, Austria). To determine fermentation characteristics, organic acids were measured in ensiled sorghum samples by gas chromatography (Varian GC 3900, Munich, Germany) according to Zhao et al. [26]. Ammonia-N concentration was determined by UV-absorption (Hitachi U5100 Spectrophotometer, Tokyo, Japan) using a commercial kit (R-Biopharm, Darmstadt, Germany). All chemical analyses were performed in duplicate.

### 2.5. Apparent Total Tract Digestibility

The ATTD of Ash, DM, OM, CP, CF, starch, EE_h_, P and Ca were calculated using the following Equation:

ATTD = [(Fi − Ff)/Fi],

ATTD = apparent total tract digestibility of DM, OM, Ash, CP, CF, starch, EE_h_, P or Ca in percent (%); Fi = total intake of Ash (g), DM (g), OM (g), CP (g), CF (g), starch (g) EE_h_ (g), P (g) or Ca (g), during the collecting period; Ff = total fecal output of Ash (g), DM (g), OM (g), CP (g), CF (g), starch (g), EE_h_ (g), P (g) or Ca (g) originating from feed that was fed during the collecting period.

Digestible energy (DE) was calculated as follows:

DE (MJ/kg DM) = [GE (MJ/kg DM) × ATTD of GE (%)]/100

The concentration of metabolizable energy (ME) was calculated by regression model based on digestible crude nutrients [23] as follows:

ME (MJ/kg) = 0.0205 × digestible protein + 0.0398 × digestible ether extract after acid hydrolysis + 0.0173 × starch + 0.0160 × sugar + 0.0147 × digestible residual.

### 2.6. Statistical Analyses

Data were analyzed as a Latin square design. Therefore, we assumed a linear mixed model, which was computed and subjected to ANOVA using the MIXED procedure of SAS 6.1 (SAS Inst., Inc., Cary, NC, USA) according to the following model: x_ijk_ = µ + T_i_ + BW + P_j_ + A_k_ + e_ijk_, where x_ijk_ = the dependent variable, µ = overall mean, T_i_ = the effect of treatments (i = sorghum with DM concentrations of 701 (S1), 738 (S2) and 809 g kg^−1^ (S3)), BW = the effect of BW at the start of the experimental period, P_j_ = the random effect of the experimental period (j = 1–3), A_k_ = the random effect of the animals (k = 1–9), and e_ijk_ = the residual experimental error.

The treatment means were separated using the least-squares means statement, and differences were determined using the Tukey–Kramer test. The differences were considered statistically significant at *p* ≤ 0.05. Results are presented as least-squares means including pooled standard error of means (SEM).

The minimum effective sample size for the experiment was estimated using the software package G*Power 3.1.9.6 [27,28]. The datasets of Humer et al. [12,13] were used to get a realistic estimation of the effect difference to calculate the minimum necessary number of animals to reach a minimum statistical power of 1-β = 0.8 at α = 0.05.

## 3. Results

### 3.1. Chemical Composition of Sorghum and Experimental Diets

Analyzed nutrient contents of ensiled sorghum grains including fermentation characteristics are shown in Table 2. Only small variations related to the nutritional and GE content were observed between the treatments. However, samples of sorghum conserves S1 and S2 contained more sugar than S3. With a DM content of 809 g kg^−1^ (S3), less acetic acid and butyric acid was generated compared to S1 and S2. On the contrary, S2 expressed the lowest concentration of lactic acid compared with S1 and S3. The amount of NH_3_ decreased with decreasing DM content, while the CP contents were similar between batches.

### 3.2. Apparent Total Tract Digestibility of Pigs

All pigs remained healthy throughout the experiment, according to continuous veterinary surveillance. Supplementary Table S1 highlights the zootechnical data obtained during the study. This was not considered for statistical analysis and data discussion, since the experimental setup was not designed for this purpose.

Results on the ATTD of energy, crude nutrients and minerals are given in Table 3. The ATTD of DM, OM, nitrogen free extract (NfE), Ash and GE was significantly higher when feeding S1 compared with S3 (*p* ≤ 0.05) while the feeding of S2 resulted in intermediate values. Feeding S3 resulted in the significantly lowest ATTD of starch compared to S1 and S2 (*p* ≤ 0.05). Furthermore, pigs fed S1 showed significantly higher ATTD of P compared with S2 and S3 (*p* ≤ 0.05). The ATTD of CP, EE_h_, CF and Ca were not significantly affected by the ensiling process.

Table 4 shows the effect of sorghum conservation on DE and ME calculated from the crude nutrient digestibility data collected during the study. The sorghum grain conserves expressed no significant differences with respect to DE and ME concentrations.

## 4. Discussion

Sorghum grain can partially replace maize in pig and poultry diets and may become increasingly important in the future due to climate change and the associated wider global distribution of maize pests like the western corn rootworm [3,29,30]. Fermentation of cereal grains under air-tight conditions requires substantially less energy than the common practice of hot air drying, thus, efficiently reducing the costs and environmental impact of feed storage as well as mycotoxin contaminations due to early harvest [31,32,33]. Furthermore, microbial pre-digestion of nutrients may result in improved nutrient digestibility and increased metabolizable energy concentration during the fermentation process [12,13]. The efficiency of the fermentation process as well as the nutrient digestibility arising thereof can be affected by the total DM concentration of the grains. These issues have been only scarcely investigated for sorghum grain with respect to pig nutrition. Therefore, the present study investigated the effects of increasing DM concentrations due to different harvested dates on the whole grain fermentation of sorghum and the subsequent ATTD of energy, crude nutrients and minerals in growing pigs.

The harvest dates affected the DM concentration and the fermentation process of whole grain sorghum. Early harvest resulted in lowest DM while harvesting 8 and 16 days later increased DM by approx. 40 and 100 g kg^−1^, respectively. Although crude nutrient and starch concentrations were comparable between different sorghum batches, NH_3_ and sugar tended to decrease with increasing DM concentration at the end of fermentation. Earlier projects on whole maize grain fermentation confirmed our results on sugar and the concentration of short chain fatty acids [12,13,34]. However, contrary to our data, Humer et al. [12,13] observed an increase in NH_3_ with increasing DM concentration. The reasons for these contradictory results cannot be explained at the moment and must be subject to future research.

Although the DM concentration was different between the sorghum conserves in the present study, the overall feed value of the final fermented products in terms of crude nutrients and starch on a dry matter basis was not affected. Nevertheless, we observed significant differences in the ATTD of GE, DM, OM, starch, ash and P when feeding the different ensiled sorghum batches, with the lowest DM silage (S1) promoting the highest obtained values and S3 fed animals exhibited the lowest ATTD values in any case. The literature on grain fermentation and its effects on feed digestibility is inconsistent and sometimes contradictory, which seems to reflect differences in substrates and fermentation conditions. Furthermore, experimental designs mostly focus on the comparison between wet ensiled and air-dried grain or the effects of feeding fermented liquid diets in comparison with dried unfermented controls. Crenshaw et al. [35] performed fattening and metabolism trials with pigs to determine the nutritional value of harvested high moisture and fermented (HMSG; 758.0 g kg^−1^ DM) vs. dried sorghum grains (DSG; 857.3 kg^−1^ DM). Diets containing HMSG resulted in improved feed conversion compared with pigs fed DSG, while feed intake and average daily gain was unaffected. Although there was no effect on DM digestibility, ensiling sorghum grain with 30% moisture improved energy intake and digestibility of energy compared with DSG. The nitrogen balance and apparent N digestibility were higher in HMSG diets compared with DSG diets. In addition, Myer et al. [36] evaluated the effect of storage conditions (high moisture vs. drying) on the nutritional value of sorghum grain in growing pigs. Similar to our experiment (S1), high moisture sorghum was harvested and anaerobically stored at 750 g kg^−1^ DM for 6 months. In accordance with our results, they observed improved ATTD of DM and energy in high moisture grain. In strong contrast to our findings, however, ATTD of CP was also improved. Finally, in accordance to our findings, Jørgensen et al. [37] and Cho et al. [38] recognized improved ATTD of DM in pigs that were fed fermented liquid diets.

A series of earlier studies applied an approach comparable to ours on the effects of whole or milled maize grain fermentation [12,13]. Humer et al. [13] investigated the effect of fermented whole maize grain with a DM concentration of 696.3 g kg^−1^ in comparison to milled fermented (711.1 g DM kg^−1^) and dried (869.6 g DM kg^−1^) maize in growing pigs under isocaloric and isonitrogenous dietary conditions. The ATTD of DM and starch was improved, and the ATTD of the ether extract was numerically increased when fermented maize was fed. The increase in digestibility led to numerically higher ATTD of the GE from fermented maize variants compared to the dried maize. In accordance with our findings, the ATTD of N as well as the N retention were not affected by the fermentation. Humer et al. [12] investigated the effects of comparable maize variants like Humer et al. [13], but in the presence or without exogenous phytase, respectively. The digestibility of DM and OM were not affected by the fermentation and CP digestibility was lower in fermented maize compared to dried maize. An increase in NH_3_ concentration in fermented maize may explain our contrasting findings on non-affected CP digestibility from different fermented sorghum batches with uniform NH_3_ levels.

Reducing the particle size of grains may also positively affect fermentation as well as GE digestibility. It is well known that storage procedures using fermentation techniques can promote microbial phytate degradation in grains, leading to improved mineral absorbability [12,13,39,40,41,42]. This could be exacerbated by the native endogenous grain phytase [43]. Our findings are in favor of a significant P release from phytate when fermenting low DM sorghum (variant S1, DM of 701 g kg^−1^). A plausible explanation could be that lactic acid bacteria decreased phytic acid contents due to their ability to produce phytase [44,45]. This is in line with data from Pieper et al. [21], who observed higher ATTD of P in pigs fed moist triticale and wheat supplemented with lactic acid bacteria compared to conventionally dried grains. Overall, our data most likely reflected microbial phytate breakdown since sorghum has a reportedly negligible native phytase activity [46].

Further research regarding the different DM concentrations of fermented sorghum grain should be carried out to identify minimal and maximal moisture levels for ideal fermentation conditions. Especially different particle sizes of sorghum grains prior to fermentation may result in varying nutrient digestibility, since several groups recognized a superior ATTD of nutrients from milled ensiled grains compared to whole grain silage [12,13,20]. However, this has yet to be proven for sorghum.

## 5. Conclusions

Our data suggest that the air-tight storage of whole grain sorghum with a DM concentration at harvest of 701 g kg^−1^ (S1) can improve the feeding value of sorghum for pigs compared to high DM variants. Especially, a lower need for the supplementation of inorganic P by 0.39 g kg^−1^ DM and reduced manure P emissions arising thereof, could enhance the sustainability of pork production based on diets with sorghum grain conserves.

## Figures and Tables

**Table 1:** Composition and calculated nutrients of test diets.

Ingredient	Amount, g kg^−1^
Sorghum	950.5
Limestone	15.5
Salt	2.5
L-Lysine-HCl	12.0
DL-Methionine	3.5
L-Threonine	4.7
L-Valine	2.0
L-Isoleucine	2.4
L-Tryptophan	1.4
Choline chloride	0.5
Vitamin and trace mineral premix ^1^	5.0
**Chemical composition**	**g kg^−1^ DM**
Dry matter	897
Crude protein	113
Ether extract	33
Ash	15
Crude fiber	25
SID Lysine	11.3
SID Methionine and Cysteine	6.2
SID Threonine	7.5
SID Tryptophan	2.2
SID Valine	5.3
SID Isoleucine	5.0
SID Leucine	9.5
SID Histidine	1.7
Calcium	6.5
Total Phosphorus	2.6
Natrium	1.1

SID, standardized ileal digestibility; ^1^ Amount per kg premix: vitamin A 1,200,000 I.E.; vitamin D_3_ 391,120 I.E.; vitamin E (as all-rac-α-tocopheryl acetate) 11,000 mg; vitamin K_3_ 840 mg, vitamin C 12,000 mg; vitamin B_1_ 480 mg; vitamin B_2_ 1200 mg, vitamin B_6_ (Pyridoxine hydrochloride) 840 mg; vitamin B_12_ 8400 μg; nicotinic acid 7200 mg; pantothenic acid 3000 mg; folic acid 120 mg; biotin 18,000 μg; iron (as iron-(II)-sulfate, monohydrate) 15,640 mg; zinc (as zinc sulfate, monohydrate) 15,640 mg; manganese (as manganese-(II)-oxide 7800 mg; copper (as copper-(II)-sulfate, pentahydrate) 2900; iodine (as calcium iodate, anhydrous) 290 mg; cobalt (as alkaline cobalt-(II)-carbonate, monohydrate) 97 mg; selenium (as sodium selenite) 88 mg; antioxidants 2930 mg; calcium carbonate as carrier (218 g kg^−1^ calcium).

**Table 2:** Analyzed nutrient composition and fermentation characteristics of different whole grain sorghum conserves stored under anaerobic conditions for 6 months.

	Experimental Group		
	S1	S2	S3
**Nutrients**			
Dry matter (g kg^−1^)	701.2	738.4	809.5
Organic matter (g kg^−1^ DM)	979.8	980.2	979.9
Crude protein (g kg^−1^ DM)	95.4	93.1	98.6
Ether extract_h_ (g kg^−1^ DM)	35.8	36.2	34.7
Crude fiber (g kg^−1^ DM)	27.5	28.6	24.9
Ash (g kg^−1^ DM)	20.2	19.8	20.1
Starch (g kg^−1^ DM)	751.4	760.9	763.0
Sugar (g kg^−1^ DM)	13.8	14.5	4.9
Gross energy (MJ kg^−1^ DM)	18.5	18.8	18.9
Phosphor (g kg^−1^ DM)	4.4	4.3	4.5
Calcium (g kg^−1^ DM)	1.5	1.6	1.5
**Fermentation characteristics**			
Acetic acid (g kg^−1^ DM)	3.3	4.1	1.6
Butyric acid (g kg^−1^ DM)	0.6	0.3	0.1
Lactic acid (g kg^−1^ DM)	4.4	1.4	3.0
NH_3_ (g kg^−1^ N)	38.1	25.7	18.4

S1, 701 g kg^−1^ DM; S2, 738 g kg^−1^ DM; S3, 809 g kg^−1^ DM; DM, dry matter.

**Table 3:** Effects of sorghum conserves with varying dry matter concentrations (701, 738 and 809 g kg^−1^) on apparent total tract digestibility (%) of energy, crude nutrients and minerals in diets for growing pigs (*n* = 9).

Item	Experimental Group			SEM	*p*-Value
	S1	S2	S3		Treat
GE, %	90.29 ^a^	89.49 ^ab^	88.87 ^b^	0.24	0.049
DM, %	91.44 ^a^	90.81 ^ab^	89.73 ^b^	0.26	0.021
OM, %	92.91 ^a^	92.31 ^ab^	91.72 ^b^	0.18	0.020
CP, %	79.97	76.85	77.53	1.11	0.443
EE_h_, %	49.19	46.06	47.32	1.09	0.490
CF, %	76.33	75.93	73.82	1.09	0.460
NfE, %	97.26 ^a^	96.73 ^ab^	96.31 ^b^	0.12	<0.001
Starch, %	99.69 ^a^	99.62 ^a^	99.48 ^b^	0.02	<0.001
Ash, %	23.36 ^a^	16.70 ^ab^	7.91 ^b^	2.42	0.035
Ca, %	68.84	70.08	64.70	1.23	0.127
P, %	62.90 ^a^	55.93 ^b^	48.00 ^b^	1.56	<0.001

GE; gross Energy; DM, dry matter; OM, organic matter; CP, crude protein; EE_h_, ether extract after acid hydrolysis; CF, cruder fiber; NfE, nitrogen free extract; Ca, calcium; P, phosphorus; S1, 701 g kg^−1^ DM; S2, 738 g kg^−1^ DM; S3, 809 g kg^−1^ DM; ^a,b^ Means within a row without a common superscript differ (*p* ≤ 0.05). SEM, Standard error of means.

**Table 4:** Effects of sorghum conserves with varying dry matter concentrations (701, 738 and 809 g kg^−1^) on the concentrations of digestible energy and metabolizable energy in diets for growing pigs (*n* = 9).

Item	Experimental Group			SEM	*p*-Value
	S1	S2	S3		Treat
DE (MJ kg^−1^ DM)	16.6	16.8	16.8	0.04	0.098
ME (MJ kg^−1^ DM)	16.2	16.1	16.1	0.04	0.201

DE, digestible energy; ME, metabolizable energy; S1, 701 g kg^−1^ DM; S2, 738 g kg^−1^ DM; S3, 809 g kg^−1^ DM; DM, dry matter. SEM, Standard error of means.

## Data Availability

Data available on request due to restrictions privacy.The data presented in this study are available on request from the corresponding author.

## Supplementary Materials

The following are available online at https://www.mdpi.com/article/10.3390/ani11051199/s1, Table S1: Effects of sorghum conserves with varying dry matter concentration (701, 738 and 809 g kg^−1^) on zootechnical performance in diets for growing pigs.
