# Supplementary material for: Fermentation of Whole Grain Sorghum (Sorghum bicolor (L.) Moench) with Different Dry Matter Concentrations: Effect on the Apparent Total Tract Digestibility of Energy, Crude Nutrients and Minerals in Growing Pigs
Source: Animals (Basel). 2021 Apr 22;11(5):1199. doi: 10.3390/ani11051199 (PMC8143492; doi:10.3390/ani11051199)
Supplement: Supplementary file 1 [file animals-11-01199-s001.zip › animals-1193049-supplementary.pdf]

animals

Article

Fermentation of Whole Grain Sorghum (*Sorghum bicolor* (L.) Moench) with Different Dry Matter Concentrations: Effect on the Apparent Total Tract Digestibility of Energy, Crude Nutrients and Minerals in Growing Pigs

Reinhard Puntigam [1,*], Julia Slama [1], Daniel Brugger [2], Karin Leitner [3], Karl Schedle [3], Gabriela Wetscherek-Seipelt [3] and Wolfgang Wetscherek [3]

Table S1. Effects of sorghum conserves with varying dry matter concentration (701, 738 and 809 g kg^{-1}) on zootechnical performance in diets for growing pigs.

Item	Experimental Group			SEM	*p*-Value
	S1	S2	S3		Treat
ADG, g d^{-1}	426.9	456.5	447.2	23.3	0.529
F:G, g kg^{-1}	3.33	2.86	2.66	0.30	0.201

S1, 701 g kg^{-1} DM; S2, 738 g kg^{-1} DM; S3, 809 g kg^{-1} DM; SEM, Standard error of means.
